# Effects of Cornelian Cherry on Microbiota in Male Professional Football Players

**DOI:** 10.3390/foods15213985

**Published:** 2026-11-06

**Authors:** Emre Batuhan Kenger, Tuğçe Özlü Karahan, Demet Yılmaz Karadag, Fatih Eren, Fatma Esra Güneş

**Affiliations:** 1 Department of Nutrition and Dietetics, Faculty of Health Sciences, Fenerbahce University, Istanbul 34758, Türkiye; emrebatuhan.kenger@gmail.com; 2 Department of Nutrition and Dietetics, Hamidiye Faculty of Health Sciences, University of Health Sciences Türkiye, Istanbul 34668, Türkiye; tugce.ozlukarahan@sbu.edu.tr; 3 Institute of Gastroenterology, Marmara University, Istanbul 34854, Türkiye; yilmz.demet@gmail.com; 4 School of Medicine, Recep Tayyip Erdogan University, Rize 53100, Türkiye; 5 Liver Research Unit, Institute of Gastroenterology, Marmara University, Istanbul 34854, Türkiye; 6 Department of Medical Biology, School of Medicine, Marmara University, Istanbul 34854, Türkiye; 7 Department of Nutrition and Dietetics, Faculty of Health Sciences, Istanbul Medeniyet University, Istanbul 34862, Türkiye; fatmaesra.gunes@medeniyet.edu.tr

**Keywords:** cornelian cherries, microbiota, football player, performance

## Abstract

This study aimed to investigate the effects of lyophilized cornelian cherry supplementation on gut microbiota composition in male professional football players aged 18–37 years recruited from a football club competing in the Turkish Football Federation Second League in Istanbul, Türkiye. The participating football players received 30 g of cornelian cherries containing 350 mg of anthocyanins for six weeks. The fecal microbiota communities of the football players were analyzed using the 16S rRNA gene sequencing. Moreover, their nutritional status, body compositions, and vertical jump performance were assessed. In the cornelian cherry group, the median relative abundance of *Parabacteroides goldsteinii* increased from 0.06% (0.01–0.17%) at baseline to 0.37% (0.11–15.44%) at week 6 (*p* = 0.005). Similarly, median body fat percentage decreased from 10.8% (5.0–16.2%) to 9.6% (4.0–14.6%) (*p* = 0.019), while median vertical jump height increased from 37.7 cm (31.9–46.5 cm) to 39.3 cm (34.6–49.0 cm) (*p* = 0.005). However, the between-group difference in the absolute change in *Parabacteroides goldsteinii* relative abundance was not statistically significant (*p* = 0.190). Cornelian cherry supplementation was not associated with significant changes in gut microbial alpha or beta diversity. However, an increase in the relative abundance of *Parabacteroides goldsteinii,* along with changes in body composition and vertical jump performance, was observed in the intervention group. These preliminary findings suggest that changes in selected microbial, anthropometric, and performance outcomes occurred during the intervention, although supplementation-specific effects cannot be established. Larger, placebo-controlled trials are warranted to confirm these observations.

## 1. Introduction

Football is among the most popular sports in the world in terms of the number of participants and spectators. Due to the popularity of football, it is vital to develop strategies to maintain and improve performance and prevent diseases and injuries [1]. As the importance of nutrition in sports increases, microbiota is one of the new research areas to emerge in this field. The human microbiota comprises complex microbial communities inhabiting various body sites, particularly the gastrointestinal tract [2]. It plays essential roles in nutrient metabolism, immune regulation, protection against pathogens, and maintenance of intestinal epithelial function [3,4,5]. Furthermore, increased gut microbial diversity, the abundance of beneficial bacterial species, and the production of short-chain fatty acids have been associated with improved physical performance [6]. The gut microbiota is influenced by several factors, including dietary habits, stress, and physical activity [7]. Moderate-intensity exercise may influence gut microbial composition, as demonstrated by Motiani et al. (2019), who reported a significant increase in *Veillonella dispar* abundance and a nonsignificant increasing trend in *Faecalibacterium prausnitzii* following moderate-intensity continuous training [8]. In contrast, prolonged and strenuous exercise may be associated with alterations in microbial composition [9,10], including a reduction in butyrate-producing bacteria such as *Faecalibacterium prausnitzii* and an increase in taxa such as *Collinsella aerofaciens*, as reported in ultramarathon runners [11]. Dietary components, particularly anthocyanin-rich fruits, may selectively modulate gut microbiota composition, with some clinical studies reporting increased abundances of specific bacterial taxa, including *Bifidobacterium* spp. and *Faecalibacterium prausnitzii* [12]. However, these microbial responses may vary depending on exercise intensity and duration, dietary factors, and individual characteristics.

*Cornus mas* L., commonly known as cornelian cherry, is an anthocyanin-rich fruit with reported antioxidant and anti-inflammatory properties [13,14]. Its bioactive constituents, particularly polyphenols and iridoids, may interact with the intestinal microbiota and undergo microbial biotransformation. Olędzka et al. (2022) demonstrated that cornelian cherry-derived compounds undergo transformations during simulated gastrointestinal digestion and ex vivo exposure to human fecal microbiota [15]. More recently, a preclinical study investigated the effects of *Cornus mas* extract on microbial composition and reported changes in specific bacterial groups, including a reduction in Enterobacteriaceae abundance [16]. These findings provide a scientific rationale for investigating the potential interactions between cornelian cherry supplementation and gut microbiota.

Despite growing evidence regarding the interactions between dietary anthocyanins and gut microbiota, the effects of cornelian cherry supplementation in professional athletes remain insufficiently investigated. Professional football players represent a relevant population because their intensive training demands may influence gastrointestinal health and microbial composition. Therefore, this study aimed to investigate the effects of six weeks of lyophilized cornelian cherry supplementation on gut microbiota composition, including alpha and beta diversity and taxonomic composition, in male professional football players. Changes in body composition and vertical jump performance were also evaluated. We hypothesized that cornelian cherry supplementation would influence gut microbial composition, potentially increasing the relative abundance of beneficial bacterial taxa.

## 2. Materials and Methods

### 2.1. Data Collection

This study was conducted with the male football players of a professional club in the Turkish Football Federation Second League and is based in Istanbul. Football players whose microbiota profile was determined in a previous study [17] were included in the present study. Prepared according to the ethical standards of the Declaration of Helsinki, ethical approval of the study was obtained from Marmara University Clinical Research Ethics Committee (no: 09.2020.952). An application was made to Bahçeşehir University Scientific Research Projects and Technology Transfer Office for the study. The study was approved with the application number BAP.2021-02.06.

Power analysis was performed during sample selection. Prevalence was calculated to be 20%, type 1 error rate to be α = 0.05, and the power of the test to be 1 − β = 0.95. The minimum number of samples per group was determined to be nine based on the power analysis. However, considering that the participants might want to leave the study and the structure of the football team, we deemed it appropriate to include 11 participants in each group.

A study investigating the effects of berry fruits on the microbiota revealed that the change in the microbiota was observed at the end of 6 weeks [18]. Hence, the present study was planned to last 6 weeks.

Twenty-three professional male football players aged 18–37 years were included in the study. They were randomly divided into two groups. The exclusion criteria included: (i) antibiotic use during the study period; (ii) use of nonsteroidal anti-inflammatory drugs (NSAIDs) that could potentially influence gut microbiota composition; (iii) gastrointestinal infections or clinically relevant gastrointestinal symptoms; and (iv) musculoskeletal injuries, particularly lower-extremity injuries that could interfere with regular training participation or affect vertical jump performance. Prior to enrollment, participants were screened for relevant medical history, medication use (including antibiotics and nonsteroidal anti-inflammatory drugs), and gastrointestinal infections or symptoms that could potentially influence gut microbiota composition. One participant in the cornelian cherry group was excluded from the study due to a cruciate ligament injury at the beginning of the intervention. Additionally, two participants in the control group were excluded from the microbiota analyses due to antibiotic use (*n* = 1) and gastrointestinal discomfort (*n* = 1), respectively. No participants in the cornelian cherry group reported antibiotic or NSAID use or gastrointestinal infections during the intervention period.

-Cornelian cherry group (*n* = 10): The football players who agreed to participate in the study were included in the cornelian cherry group using random sampling. Without interfering with their regular diet, the participants in this group were given 350 mg of anthocyanin-containing lyophilized cornelian cherries once a day in addition to their regular diet.

-Control group (*n * = 12): The football players who agreed to participate in the study were included in the control group using random sampling. The diet of the participants in this group was not interfered with.

The groups were matched according to the players’ position (goalkeeper, fullback, etc.). The nutritional status of the football players was found by showing them the food atlas in person and taking a 3-day food consumption record. The food consumption records were analyzed with Nutrition Information Systems (BeBiS) Version 8.1 (Pasific Ltd. Şti., Istanbul, Turkey). Voluntary consent was obtained from all participants in the beginning.

The participants in both groups were interviewed face-to-face every week. The fruit consumption status of those in the cornelian cherry group and the dietary habits of those in the control group were questioned. The participants who were assigned to receive cornelian cherries were given 30 g of lyophilized cornelian cherries (containing 350 mg of anthocyanin) packed in vacuum packs during the face-to-face interviews. At the beginning and end of the study, the participants’ performance was measured, their anthropometric measurements were made, and fecal samples were collected for gut microbiota analysis.

### 2.2. Anthropometric Measurements

Anthropometric variables included height, body weight, body mass index (BMI), body fat percentage, and muscle mass. Body height was measured with an accuracy of 0.1 cm using the Harpenden anthropometer (Holtain Ltd., Croswell, UK). Body composition was determined using a bioelectrical impedance analyzer called Inbody 120R. The anthropometric measurements were carried out on an empty stomach at the beginning and end of the study in the same environment in the morning. The players were asked not to eat or drink before the measurement process. It was ensured that they dressed lightly (in shorts), and any metal items, such as watches and necklaces, were removed before stepping on the device. The anthropometric measurements were planned to take place the day after the day when they did not train.

### 2.3. Performance Tests

OptoJump NextR (version 1.10, Microgate, Bolzano, Italy), developed by Micro Gate, was used to measure vertical jumps. A validity and reliability study was conducted to determine the vertical jump height of the device. The participants were asked to jump vertically between the OptoJump bars three times in a row, with their feet parallel to the bars. They were asked to reach the maximum height during this jump [19]. The jump height of the participants was calculated in centimeters (cm) thanks to the sensors of the device. The participants were asked to have their hands free. The highest jump height measured was used as the height value [20].

### 2.4. Microbiota Analysis

Gut microbiota analyses were conducted at baseline and following the six-week intervention using the methodology previously described by Kenger et al. [17].

Fecal samples were collected from the participants at the football club facilities by a member of the research team at baseline and at the end of the six-week intervention. Following collection, the samples were transferred into DNA/RNA Shield™ preservation solution (Zymo Research, Irvine, CA, USA) and transported to the laboratory on dry ice. Upon arrival, the samples were stored at −86 °C until DNA extraction. Bacterial DNA was extracted from the preserved fecal samples using the ZymoBIOMICS™ DNA Miniprep Kit (Zymo Research, Irvine, CA, USA), according to the manufacturer’s instructions. The optical densities of the isolated DNA samples were measured at 260 nm using the Qubit dsDNA Broad Range (BR) Assay Kit and a Qubit fluorometer (Thermo Fisher Scientific, Waltham, MA, USA), and the DNA concentrations were determined. Based on the obtained values, all DNA samples were adjusted to a final concentration of 10 ng/µL to standardize the initial DNA concentrations for subsequent polymerase chain reaction (PCR) amplification. Additionally, DNA extraction quality was evaluated using 0.5% agarose gel electrophoresis to assess DNA integrity and potential contamination. In cases of insufficient or inappropriate DNA extraction, the extraction procedure was repeated.

The V3–V4 hypervariable regions of the bacterial 16S rRNA gene were amplified by PCR using universal bacterial forward and reverse primers. These primers were selected based on the comprehensive evaluation conducted by Klindworth et al., which identified effective primer sets for bacterial 16S rRNA gene amplification. A two-step PCR protocol was employed for sequencing library preparation, and KAPA HiFi HotStart ReadyMix was used in both amplification steps. Following the initial amplicon PCR, the PCR products were visualized by agarose gel electrophoresis using a 100 bp DNA ladder. PCR products exhibiting the expected target fragment size without nonspecific amplification bands were purified using the magnetic bead-based Sera-Mag Select system. Following index PCR, the amplified products were purified using the same magnetic bead-based approach, and the concentrations of double-stranded DNA products were measured fluorometrically using the Qubit dsDNA High Sensitivity Assay Kit. Individual sequencing libraries that passed quality control were normalized to equal molar concentrations and pooled into a single tube. The pooled library was adjusted to a final concentration of 30–50 pM before loading onto the next-generation sequencing platform.

Sequencing was performed using the Illumina iSeq next-generation sequencing platform (Illumina, San Diego, CA, USA), according to the manufacturer’s instructions for the iSeq i1 Reagent Kit [21]. The sequencing process generated more than 100,000 reads per sample. Taxonomic classification was performed using the Illumina BaseSpace 16S Metagenomics workflow. Species-level assignments were generated by ClassifyReads, an extension of the naïve Bayesian RDP classification approach that supports species-level annotation and resolves ambiguous classifications at higher taxonomic ranks. These assignments were retained for exploratory analyses and interpreted as putative species-level annotations, given the limited ability of partial 16S rRNA gene sequences to distinguish some closely related species. Microbial profiles and diversity outputs were generated using the Illumina BaseSpace 16S Metagenomics application (v1.1.0). Taxonomic profiles were obtained from the sample-level classification outputs and expressed as relative abundances at the phylum, genus, and species levels. Alpha diversity was assessed using the Shannon index calculated from species-level classifications. Between-sample differences in microbial composition were explored using principal coordinate analysis (PCoA). According to the application documentation, PCoA was generated by classical multidimensional scaling of a Pearson correlation distance matrix (1 − *r*), calculated from L1-normalized sample classification vectors after excluding unclassified categories.

### 2.5. Data Analysis

Statistical analyses were performed using SPSS software (version 30.0). The individual football player was considered the experimental unit. Measurements obtained from the same participant at baseline and after the six-week intervention were treated as paired observations. The analyses included 22 participants for anthropometric and performance outcomes (cornelian cherry group, *n* = 10; control group, *n* = 12) and 20 participants for gut microbiota outcomes (*n* = 10 per group).

Considering the relatively small sample size and the skewed distributions of several variables, particularly microbial relative abundances, nonparametric statistical methods were employed. Continuous variables were summarized as medians and minimum–maximum values, while categorical variables were presented as frequencies and percentages. Within-group comparisons between baseline and week 6 were performed using the Wilcoxon signed-rank test. Absolute changes were calculated individually as the absolute difference between week 6 and baseline values (|Δ| = |week 6 − baseline|), and between-group comparisons of these absolute changes were performed using the Mann–Whitney U test. Exploratory associations between changes in selected bacterial taxa, mean dietary intake, body composition, and physical performance were assessed using Spearman’s rank correlation coefficients. Microbiota-related visualizations were generated using ggplot. Statistical significance was set at *p* < 0.05.

## 3. Results

Twenty-two professional football players aged 18–37 years participated in the study. Of the participants, 27.3% were professional for <5 years, and 72.7% for ≥5 years. Of them, 77.3% were high school graduates, and 22.7% had a bachelor’s degree. All the football players (100%) have regular bowel movements daily. The general characteristics of the football players are shown in Table 1.

The anthropometric measurements and physical performance outcomes of the football players at baseline and after the six-week intervention are presented in Table 2. Within the cornelian cherry group, median fat mass decreased significantly from 7.3 to 6.4 kg (*p* = 0.016), and median body fat percentage decreased from 10.8% to 9.6% (*p* = 0.019). Additionally, median vertical jump height increased significantly from 37.7 to 39.3 cm (*p* = 0.005). No statistically significant within-group changes were observed in the control group (all *p* > 0.05). When the magnitudes of individual changes were compared between groups, the cornelian cherry group exhibited significantly greater absolute changes in body weight (1.1 vs. 0.2 kg), BMI (0.3 vs. 0.06 kg/m^2^), fat mass (0.9 vs. 0.1 kg), body fat percentage (1.2 vs. 0.1 percentage points), and vertical jump height (2.3 vs. 0.3 cm; all *p* < 0.001). The absolute change in jump power was also significantly greater in the cornelian cherry group (1.3 vs. 0.2 W; *p* = 0.002), although the within-group change in jump power was not statistically significant (*p* = 0.074). No statistically significant between-group difference in the absolute change in muscle mass was detected (*p* = 0.080).

Table 3 presents the estimated daily energy and nutrient intakes of the football players in the cornelian cherry and control groups at baseline and after the six-week intervention, based on three-day dietary records. The median energy, carbohydrate, protein, fat, and fiber consumption of the cornelian cherry and control groups did not change significantly during the study (*p* > 0.05).

**Figure 1:**
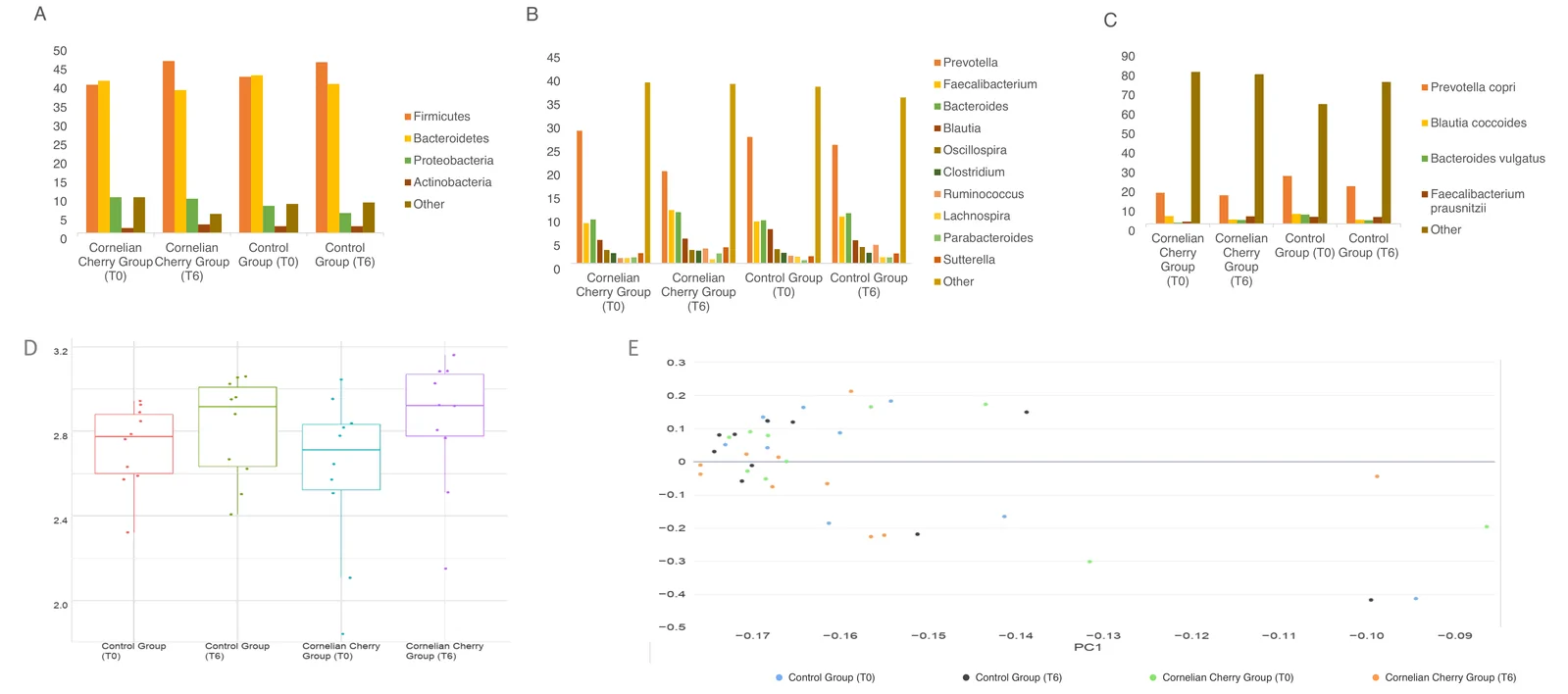
Relative abundances are presented at the phylum (**A**), genus (**B**), and species (**C**) levels; colors identify the microbial taxa listed in the corresponding legends. Panel (**D**) shows the Shannon diversity index for the Control Group at T0 (red) and T6 (green) and the Cornelian Cherry Group at T0 (turquoise) and T6 (purple). Panel (**E**) shows the principal coordinate analysis, with the Control Group at T0 shown in blue and at T6 in black and the Cornelian Cherry Group at T0 shown in green and at T6 in orange. Individual points in panels D and E represent individual samples. T0 and T6 indicate the study time points.

Changes in gut microbiota composition at the phylum, genus, and species levels, together with alpha and beta diversity measures, are presented in Figure 1A–E. Twenty-six phyla of intestinal microbiota were detected in the cornelian cherry and control groups at the beginning of the study. At the phylum level, Firmicutes, Bacteroidetes, Proteobacteria, and Actinobacteria were the predominant bacterial phyla in both groups, each exhibiting a relative abundance greater than 1%. No statistically significant changes in the relative abundances of these phyla were observed in either the cornelian cherry or control group following the six-week intervention (*p* > 0.05). At the genus level, ten bacterial genera with relative abundances exceeding 1% were identified in both groups: *Prevotella, Bacteroides, Faecalibacterium, Blautia, Oscillospira, Clostridium, Sutterella, Ruminococcus, Lachnospira*, and *Parabacteroides*. No statistically significant changes in the relative abundances of these genera were observed within either group during the intervention period (*p* > 0.05). At the species level, four bacterial species with relative abundances exceeding 1% were identified in both groups: *Prevotella copri, Blautia coccoides, Bacteroides vulgatus, and Faecalibacterium prausnitzii*. No statistically significant changes in the relative abundances of these species were observed within either the cornelian cherry or control group following the six-week intervention (all *p* > 0.05).

When the groups were evaluated using the Shannon index, there was no statistically significant difference between the groups at the beginning and end of the study (*p* = 0.63). There was no significant difference in the microbiota of the football players who received cornelian cherry supplementation in terms of Shannon index at the end of 6 weeks (*p* = 0.123). No significant difference was found in the microbiota in terms of Shannon index in the control group at the end of 6 weeks (*p*: 0.247). Additionally, there was no significant difference in the coordinate analysis between the groups.

Five bacterial species exhibiting nominally significant within-group changes in at least one study group were selected for further exploratory analysis. Changes in the relative abundance of selected bacterial species during the six-week intervention are presented in Table 4. In the cornelian cherry group, the median relative abundance of *Parabacteroides goldsteinii* increased significantly from 0.06% at baseline to 0.37% at week 6 (*p* = 0.005), whereas no statistically significant within-group change was observed in the control group (0.02% to 0.07%; *p* = 0.170). In the control group, a significant increase in the relative abundance of *Clostridium taeniosporum* was observed (*p* = 0.005), while significant decreases were detected in *Streptomyces lazureus* (*p* = 0.007), *Prevotella pleuritidis* (*p* = 0.005), and *Bdellovibrio exovorus* (*p* = 0.005). However, between-group comparisons revealed no statistically significant differences in the magnitudes of absolute changes for *Parabacteroides goldsteinii* (*p* = 0.190), *Clostridium taeniosporum* (*p* = 0.089), *Streptomyces lazureus* (*p* = 0.315), *Prevotella pleuritidis* (*p* = 1.000), or *Bdellovibrio exovorus* (*p* = 0.190).

Exploratory Spearman correlation analyses were performed to examine associations between changes in the relative abundance of five selected bacterial species and mean dietary intake, body composition, and physical performance (Table 5). Changes in *Streptomyces lazureus* were positively correlated with mean fat intake (ρ = 0.514, *p* = 0.020) and changes in muscle mass (ρ = 0.515, *p* = 0.020). Similarly, changes in *Bdellovibrio exovorus* were positively correlated with mean energy intake (ρ = 0.456, *p* = 0.043) and mean fat intake (ρ = 0.490, *p* = 0.028).

## 4. Discussion

Prolonged and strenuous exercise may be associated with gastrointestinal disturbances in some athletes, although these responses can vary depending on exercise intensity, duration, and individual characteristics. Considering the potential interactions between anthocyanin-rich foods and gut microbiota, the present study investigated changes in gut microbiota composition, body composition, and vertical jump performance following six weeks of cornelian cherry supplementation in male professional football players.

In the present study, a statistically significant within-group increase in vertical jump height was observed among football players receiving cornelian cherry supplementation following the six-week intervention (*p* = 0.005), whereas the change in jump power was not statistically significant (*p* > 0.05). To our knowledge, previous studies have not specifically investigated the effects of cornelian cherry supplementation on physical performance in professional football players. However, studies examining other anthocyanin-rich fruits have reported inconsistent findings. For example, a randomized controlled trial involving semi-professional football players reported improved jump performance during recovery following Montmorency tart cherry supplementation [22]. In contrast, another study involving professional football players found no significant differences in jump height following tart cherry juice consumption [23]. Similarly, New Zealand blackcurrant supplementation did not significantly affect jump power in a previous investigation [24]. These differences may reflect variations in the phytochemical composition of the supplemented fruits, intervention duration, study populations, and performance assessment protocols. Previous studies have proposed that anthocyanin-rich foods may influence exercise performance and recovery through vascular, antioxidant, and anti-inflammatory pathways [25,26,27]. However, these mechanisms were not directly investigated in the present study. Therefore, the observed improvement in vertical jump height should be interpreted cautiously.

A statistically significant within-group reduction in body fat percentage was also observed in the cornelian cherry group following the six-week intervention. Previous studies investigating anthocyanin-containing supplements and fruit products have reported varying effects on body composition. While some investigations have demonstrated reductions in body fat mass or percentage, others have reported changes that did not reach statistical significance [28,29,30]. Potential mechanisms discussed in the literature include the regulation of lipid metabolism and energy balance through pathways involving lipid synthesis and AMP-activated protein kinase (AMPK) signaling [31,32]. Nevertheless, these mechanisms remain speculative in the context of the present study, as relevant biochemical and molecular markers were not assessed. Furthermore, cornelian cherry contains several bioactive constituents in addition to anthocyanins, making it difficult to identify the contribution of individual compounds. Consequently, the observed changes in body composition should not be interpreted as direct evidence of anthocyanin-mediated metabolic effects. Further controlled studies are required to determine whether cornelian cherry supplementation produces measurable effects on physical performance and body composition beyond those associated with training, habitual dietary intake, and other individual factors.

When the effects of cornelian cherry consumption on the gut microbiota of football players were examined, no statistically significant changes were observed in Shannon alpha diversity or beta diversity over the six-week study period. However, a statistically significant within-group increase in the relative abundance of *Parabacteroides goldsteinii* was observed in the cornelian cherry group (*p* = 0.005). Previous experimental studies have suggested that fruit-derived bioactive compounds may influence the abundance and growth of *Parabacteroides goldsteinii*. Medina-Larqué et al. (2022) reported an increased relative abundance of *Parabacteroides goldsteinii* following cranberry polyphenol supplementation in mice fed an obesogenic diet [33]. More recently, Song et al. (2026) reported that supplementation with Schisandra chinensis, a fruit containing various bioactive compounds, enriched *Parabacteroides goldsteinii* in a mouse model of experimental colitis [34]. Although these studies involved different fruits and experimental models, their findings provide preliminary evidence that certain fruit-derived bioactive compounds may influence *Parabacteroides goldsteinii*. Previous research has also suggested potential associations between *Parabacteroides goldsteinii* and inflammatory and metabolic parameters, including insulin resistance and obesity-related outcomes [35]. Based on its potential health-related properties, *Parabacteroides goldsteinii* has been proposed as a candidate next-generation probiotic [36]. Considering that cornelian cherry contains anthocyanins and other polyphenolic compounds, these constituents may have contributed to the observed microbial change. However, this explanation remains hypothetical, as the individual contributions of these compounds, microbial metabolites, and associated metabolic pathways were not directly investigated in the present study. Furthermore, exploratory correlation analyses revealed no statistically significant associations between changes in *Parabacteroides goldsteinii* relative abundance and mean dietary energy, protein, fat, or carbohydrate intake (all *p* > 0.05). Similarly, no significant correlations were identified with changes in fat mass (ρ = 0.011, *p* = 0.965), muscle mass (ρ = −0.037, *p* = 0.876), vertical jump height (ρ = 0.325, *p* = 0.163), or jump power (ρ = 0.190, *p* = 0.423). These findings suggest that the observed variation in *Parabacteroides goldsteinii* relative abundance was not demonstrably associated with nutritional intake, body composition, or physical performance outcomes in the present cohort.

Beyond the observed changes in *Parabacteroides goldsteinii*, previous studies investigating anthocyanin-rich fruits have reported changes in other bacterial taxa, providing a broader context for interpreting the microbial responses observed in the present study. A previous study investigating the effects of blueberry juice containing 375 mg of anthocyanins for six weeks reported a significant increase in *Bifidobacterium longum subsp. infantis* abundance in individuals with cardiovascular risk factors [18]. In contrast, no statistically significant change in the relative abundance of *Bifidobacterium* was detected in the present study, despite supplementation with cornelian cherry providing a comparable anthocyanin dose (350 mg/day). This discrepancy may be explained by differences in the phytochemical composition and bioavailability of anthocyanins between blueberry juice and lyophilized cornelian cherry, as well as variations in the baseline gut microbiota composition and physiological characteristics of the study populations. Furthermore, a recent study with experimental diabetic mice revealed that the consumption of cornelian cherry and inulin together led to an increase in *Bifidobacterium* in the microbiota. The study indicated that the use of cornelian cherry and inulin together could be a functional prebiotic food [37]. Similarly, an in vitro gastrointestinal digestion study reported favorable changes in gut microbial composition following exposure to fermented cornelian cherry [38]. These findings suggest that the formulation and processing of cornelian cherry, as well as its combination with other prebiotic components, may influence microbial responses. However, further studies are needed to determine whether these factors account for the differences observed across studies.

At the end of 6 weeks, the relative abundance of *Clostridium taeniosporum* increased, and the relative abundance of *Bdellovibrio exovorus, Streptomyces lazureus*, and *Prevotella pleuritidis* decreased in the microbiota in the control group. After reviewing the literature, no study was found investigating the effects of an increase or decrease in the relative abundance of *Clostridium taeniosporum, Bdellovibrio exovorus, Streptomyces lazureus*, and *Prevotella pleuritidis* in the intestinal microbiota on human health. In particular, the reduction in the relative abundance of *Prevotella pleuritidis* in the control group may reflect natural temporal fluctuations in gut microbiota composition, interindividual microbial variability, or differences in habitual dietary patterns.

The present study has several strengths, including its focus on professional football players, the comprehensive characterization of gut microbiota composition across different taxonomic levels, and its six-week longitudinal intervention design. Furthermore, recruiting participants from the same football team helped minimize variability in training patterns, while the study groups were comparable in terms of anthropometric characteristics and playing positions. Weekly face-to-face interviews also facilitated the monitoring of participants throughout the intervention. Nevertheless, several limitations should be acknowledged. First, biochemical parameters were not assessed, limiting our ability to explore potential relationships between gut microbiota, physical performance, and biochemical markers. Second, the absence of a standardized dietary run-in period may have introduced dietary confounding. Although participants’ habitual dietary intake was monitored through food consumption records and weekly interviews, interindividual differences in baseline gut microbiota composition and dietary habits may have influenced the observed microbial responses. Additionally, the microbiota analyses included only 20 participants (10 per group), which limits the precision of the findings and may have reduced the ability to detect diet-related differences in microbial diversity, community composition, and correlations with other outcomes. Furthermore, the relatively wide age range of the participants (18–37 years) may have contributed to interindividual variability in gut microbiota composition. Therefore, the findings should be interpreted cautiously, and future studies with larger sample sizes, more rigorous dietary standardization, and appropriate longitudinal statistical approaches are warranted to better distinguish supplementation-related effects from interindividual variability.

## 5. Conclusions

This study provides preliminary evidence regarding changes in gut microbiota composition, body composition, and physical performance during six weeks of lyophilized cornelian cherry supplementation in male professional football players. No statistically significant changes were observed in Shannon alpha diversity or beta diversity (*p* > 0.05). However, the median relative abundance of *Parabacteroides goldsteinii* increased from 0.06% to 0.37% in the cornelian cherry group (*p* = 0.005), whereas the corresponding change in the control group (0.02% to 0.07%) was not statistically significant (*p* = 0.170).

Regarding body composition and physical performance, the cornelian cherry group exhibited a significant within-group reduction in median body fat percentage from 10.8% to 9.6% (*p* = 0.019) and fat mass from 7.3 to 6.4 kg (*p* = 0.016). Additionally, median vertical jump height increased from 37.7 to 39.3 cm (*p* = 0.005), while the change in jump power was not statistically significant (*p* = 0.074). No significant within-group changes in these outcomes were observed in the control group.

These findings highlight changes in specific microbial taxa, body composition, and vertical jump performance that warrant further investigation. Nevertheless, the observed within-group differences do not establish a supplementation-specific effect or demonstrate that changes in gut microbiota contributed to improvements in body composition or physical performance. Although a statistically significant within-group increase in the relative abundance of *Parabacteroides goldsteinii* was observed in the cornelian cherry group, the between-group difference in the magnitude of change was not statistically significant. Therefore, this finding cannot be considered evidence of a supplementation-specific effect and requires confirmation in larger, placebo-controlled trials with rigorous dietary control.

## Figures and Tables

**Table 1:** Distribution of general characteristics of football players.

	Count (n)	Percentage (%)
**Marial status**		
Married	8	36.4
Single	14	63.6
**Educational status**		
High school graduate	17	77.3
Graduate	5	22.7
**Years of professional experience**		
<5 years	6	27.3
≥5 years	16	72.7
**Regular use of medication**		
No	22	100.0
**Regular sleep pattern**		
Yes	20	90.9
No	2	9.1
**Regular bowel movements**		
Yes	22	100.0

**Table 2:** Anthropometric measurements and physical performance outcomes at baseline and after the six-week intervention.

Parameter	Group	Baseline Median (Min-Max)	Week 6 Median (Min-Max)	Within-Group *p*	Absolute Change |Δ| Median (Min-Max)	Between-Group *p* for |Δ|
**Body Weight (kg)**	**Cornelian Cherry (n = 10)**	70.6 (64.0–88.5)	71.2 (62.9–86.7)	0.332	1.1 (0.2–1.9)	**<0.001**
	**Control (n = 12)**	77.2 (61.2–83.1)	76.9 (61.3–82.4)	0.495	0.2 (0.1–0.5)	
**BMI (kg/m^2^)**	**Cornelian Cherry (n = 10)**	23.4 (20.6–25.1)	23.6 (20.3–25.5)	0.445	0.3 (0.1–0.6)	**<0.001**
	**Control (n = 12)**	22.9 (19.3–26.0)	22.8 (19.5–26.1)	0.308	0.06 (0.01–0.16)	
**Fat mass (kg)**	**Cornelian Cherry (n = 10)**	7.3 (3.5–10.7)	6.4 (2.2–9.7)	**0.016**	0.9 (0.3–1.9)	**<0.001**
	**Control (n = 12)**	8.1 (4.0–9.8)	7.9 (4.4–10.3)	0.125	0.1 (0.1–0.3)	
**Fat %**	**Cornelian Cherry (n = 10)**	10.8 (5.0–16.2)	9.6 (4.0–14.6)	**0.019**	1.2 (0.3–2.2)	**<0.001**
	**Control (n = 12)**	10.9 (5.8–12.4)	10.7 (6.2–13.5)	0.711	0.1 (0.02–0.1)	
**Muscle mass (kg)**	**Cornelian Cherry (n = 10)**	36.8 (31.4–45.1)	37.1 (32.0–45.7)	0.138	0.2 (0.1–1.3)	0.080
	**Control (n = 12)**	40.1 (32.8–42.6)	40.4 (31.9–41.8)	0.936	0.1 (0.02–0.2)	
**Jump height (cm)**	**Cornelian Cherry (n = 10)**	37.7 (31.9–46.5)	39.3 (34.6–49.0)	**0.005**	2.3 (0.2–4.6)	**<0.001**
	**Control (n = 12)**	44.3 (38.2–50.1)	44.7 (37.3–49.1)	0.062	0.3 (0.2–1.2)	
**Power (W)**	**Cornelian Cherry (n = 10)**	14.5 (12.8–16.8)	15.3 (13.5–19.4)	0.074	1.3 (0.2–3.4)	**0.002**
	**Control (n = 12)**	17.8 (15.2–25.8)	18.4 (14.5–26.3)	0.874	0.2 (0.1–0.9)	

Within-group comparisons between baseline and week 6 were performed using the Wilcoxon signed-rank test. Absolute change (|Δ|) was calculated individually as |week 6 value − baseline value|. Between-group comparisons of absolute changes were performed using the Mann–Whitney U test. Statistically significant *p*-values (*p* < 0.05) are presented in bold.

**Table 3:** Estimated daily energy and nutrient intakes of the cornelian cherry and control groups at baseline and after the six-week intervention.

	Cornelian Cherry Group (n = 10)			Control Group (n = 12)		
	Baseline	Week 6		Baseline	Week 6	
	Median (Min-Max)	Median (Min-Max)	*p*	Median (Min-Max)	Median (Min-Max)	*p*
**Energy (kcal)**	2949.5 (2820.2–3131.1)	2870.9 (2684.6–3216.4)	0.235	3127.5 (2859.6–3197.0)	2980.8 (2732.9–3262.9)	0.223
**Carbohydrate (gram)**	364.5 (331.3–396.6)	368.9 (334.2–400.44)	0.333	384.1 (339.6–398.0)	382.5 (341.4–395.8)	0.182
**Carbohydrate (%)**	50.5 (48.0–52.0)	51.0 (47.0–53.0)	0.312	50.0 (46.0–52.0)	49.5 (46.0–51.5)	0.334
**Carbohydrate g/kg**	5.1 (4.8–5.3)	5.3 (4.6–5.9)	0.333	4.8 (4.5–5.6)	4.6 (4.2–5.4)	0.480
**Fiber (g)**	33.2 (24.0–39.2)	34.8 (23.2–37.6)	0.799	31.9 (25.3–39.8)	33.4 (23.9–37.0)	0.906
**Protein (g)**	139.6 (132.7–156.8)	137.9 (131.2–159.0)	0.386	149.4 (136.9–157.7)	147.5 (134.2–155.0)	0.480
**Protein (%)**	19.5 (18.0–21.0)	19.0 (17.5–21.0)	0.241	20.0 (18.0–21.0)	19.5 (17.5–21.0)	0.325
**Protein g/kg**	2.0 (1.5–2.1)	1.9 (1.4–2.3)	0.508	1.9 (1.7–2.3)	1.8 (1.6–2.1)	0.182
**Isoleucine (g)**	6.7 (6.2–7.5)	7.1 (5.4–8.1)	0.959	7.1 (6.3–7.5)	6.4 (5.9–6.8)	0.388
**Leucine (g)**	10.3 (9.8–11.2)	9.7 (8.8–12.2)	0.959	11.1 (9.9–11.8)	10.3 (8.7–10.8)	0.657
**Valine (g)**	7.4 (6.8–8.0)	6.9 (6.3–7.9)	0.799	7.8 (7.1–8.3)	6.1 (5.8–8.4)	0.695
**Fat (g)**	101.1 (87.2–107.9)	102.7 (90.0–110.3)	0.203	106.1 (95.8–116.3)	108.6 (93.1–119.5)	0.732
**Fat (%)**	30.0 (27.0–33.0)	31.0 (28.0–33.0)	0.359	31.0 (28.0–32.0)	31.5 (28.5–33.0)	0.695
**Saturated fat (g)**	40.7 (36.7–47.0)	43.0 (38.1–49.3)	0.508	44.1 (41.9–48.2)	45.4 (39.9–50.1)	0.906

Data are presented as median (minimum–maximum). Within-group comparisons between baseline and week 6 were performed using the Wilcoxon signed-rank test.

**Table 4:** Changes in the relative abundance of selected bacterial species and between-group comparisons of absolute changes during the six-week intervention in male professional football players.

Parameter	Group	Baseline Relative Abundance (%) Median (Min-Max)	Week 6 Relative Abundance (%) Median (Min-Max)	Within-Group *p*	Absolute Change |Δ| Median (Min-Max)	Between-Group *p* for |Δ|
*Parabacteroides goldsteinii*	**Cornelian Cherry (n = 10)**	0.06 (0.01–0.17)	0.37(0.11–15.44)	**0.005**	0.2598 (0.05–15.37)	0.190
	**Control (n = 10)**	0.02 (0.00–0.08)	0.07 (0.02–0.63)	0.170	0.2113 (0.00–0.58)	
*Clostridium taeniosporum*	**Cornelian Cherry (n = 10)**	0.0136 (0.0031–0.0298)	0.0435 (0.0109–0.0908)	0.354	0.0257 (0.00–0.08)	0.089
	**Control (n = 10)**	0.0182 (0.0071–0.0321)	0.0589 (0.0361–0.1082)	**0.005**	0.0402 (0.01–0.10)	
*Streptomyces lazureus*	**Cornelian Cherry (n = 10)**	0.0057 (0.0015–0.0184)	0.0011 (0.0000–0.0036)	0.115	0.0050 (0.00–0.01)	0.315
	**Control (n = 10)**	0.0103 (0.0000–0.0260)	0,0008 (0.0004–0.0042)	**0.007**	0.0082 (0.00–0.03)	
*Prevotella pleuritidis*	**Cornelian Cherry (n = 10)**	0.0406 (0.0104–0.0670)	0.0071 (0.0015–0.0210)	0.082	0.0310 (0.00–0.06)	1.000
	**Control (n = 10)**	0.0435 (0.0192–0.0765)	0.0095 (0.0042–0.0215)	**0.005**	0.0279 (0.00–0.07)	
*Bdellovibrio exovorus*	**Cornelian Cherry (n = 10)**	0.0125 (0.0015–0.0907)	0.0053 (0.0000–0.0202)	0.093	0.0046 (0.00–0.09)	0.190
	**Control (n = 10)**	0.0467 (0.0111–0.1212)	0.0086 (0.0010–0.0142)	**0.005**	0.0358 (0.00–0.11)	

Baseline and week 6 values represent bacterial relative abundances (%) and are presented as medians (minimum–maximum). Within-group comparisons between baseline and week 6 were performed using the Wilcoxon signed-rank test. Absolute change (|Δ|) was calculated individually as |week 6 value − baseline value|. Between-group comparisons of absolute changes were performed using the Mann–Whitney U test. Statistically significant *p*-values (*p* < 0.05) are presented in bold.

**Table 5:** Exploratory Spearman correlations between changes in selected bacterial species and dietary intake, body composition, and physical performance in professional male football players.

	Mean Dietary Intake			
**Bacterial Species**	**Energy (kcal)**	**Protein (g)**	**Fat (g)**	**CHO (g)**
*Parabacteroides goldsteinii*	−0.281 (0.230)	0.086 (0.719)	−0.314 (0.177)	−0.221 (0.349)
*Clostridium taeniosporum*	0.347 (0.133)	0.206 (0.384)	0.391 (0.088)	0.119 (0.618)
*Streptomyces lazureus*	0.185 (0.435)	0.042 (0.860)	0.514 **(0.020)**	−0.123 (0.605)
*Prevotella pleuritidis*	0.123 (0.605)	0.101 (0.673)	0.292 (0.212)	−0.050 (0.835)
*Bdellovibrio exovorus*	0.456 **(0.043)**	0.117 (0.622)	0.490 **(0.028)**	0.283 (0.227)
	**Body composition, and performance**			
**Bacterial species**	**Jump height Δ (cm)**	**Jump power (W) Δ**	**Fat mass Δ (kg)**	**Muscle mass Δ (kg)**
*Parabacteroides goldsteinii*	0.325 (0.163)	0.190 (0.423)	0.011 (0.965)	−0.037 (0.876)
*Clostridium taeniosporum*	−0.375 (0.103)	0.011 (0.965)	0.144 (0.545)	0.094 (0.694)
*Streptomyces lazureus*	−0.232 (0.325)	0.105 (0.658)	0.218 (0.355)	0.515 **(0.020)**
*Prevotella pleuritidis*	0.009 (0.970)	0.248 (0.291)	0.272 (0.246)	0.200 (0.399)
*Bdellovibrio exovorus*	−0.148 (0.535)	0.113 (0.635)	0.295 (0.206)	0.241 (0.305)

Values are Spearman’s rank correlation coefficients (ρ), with corresponding *p*-values in parentheses. Both correlation coefficients and *p*-values are dimensionless; units shown in the column headings refer to the underlying variables. Dietary variables represent mean daily intakes, whereas body composition and performance variables represent changes between baseline and week 6. CHO: carbohydrate. Statistically significant *p*-values (*p* < 0.05) are presented in bold.

## Data Availability

The data obtained in this study are available from the corresponding author upon request. The data are not publicly available due to subjects’ privacy.

## Abbreviations

The following abbreviations are used in this manuscript:

BMI	Body mass index
AMPK	AMP protein kinase
COPD	Chronic obstructive pulmonary disease
TNF-α	Tumor necrosis factor alpha
